# A new approach to prevent, diagnose, and treat hepatitis B in Africa

**DOI:** 10.1186/s44263-023-00026-1

**Published:** 2023-11-02

**Authors:** C. Wendy Spearman, Monique I. Andersson, Bisi Bright, Pantong M. Davwar, Hailemichael Desalegn, Alice Nanelin Guingane, Asgeir Johannessen, Kenneth Kabagambe, Maud Lemoine, Philippa C. Matthews, Gibril Ndow, Nicholas Riches, Yusuke Shimakawa, Roger Sombié, Alexander J. Stockdale, Jantjie J. Taljaard, Michael J. Vinikoor, Gilles Wandeler, Edith Okeke, Mark Sonderup, on behalf of the Hepatitis B in Africa Collaborative Network (HEPSANET)

**Affiliations:** 1 Division of Hepatology, Department of Medicine, Faculty of Health Sciences, University of Cape Town, Cape Town, South Africa; 2 Oxford University Hospitals NHS Foundation Trust, Oxford, UK; 3 Radcliffe Department of Medicine, University of Oxford, Oxford, UK; 4 Division of Medical Virology, University of Stellenbosch, Stellenbosch, South Africa; 5 LiveWell Initiative, Yesuf Abiodun Street, Victoria Island, Lagos, Nigeria; 6 Women in Hepatitis Africa, Womens Wellness Center for Hepatitis, Isale Ajoke, Iwaya-Makoko, Lagos State, Nigeria; 7 Department of Internal Medicine, Jos Univeristy Teaching Hospital, Jos, Nigeria; 8 Department of Internal Medicine, St. Paul’s Hospital Millennium Medical College, Addis Ababa, Ethiopia; 9 Hepato-Gastroenterology Department, Bogodogo University Hospital Center, Ouagadougou, Burkina Faso; 10 Department of Infectious Diseases, Vestfold Hospital Trust, Tønsberg, Norway; 11 Institute of Clinical Medicine, University of Oslo, Oslo, Norway; 12 The National Organisation for People Living With Hepatitis B, Kampala, Uganda; 13 Department of Metabolism, Digestion and Reproduction, Division of Digestive Diseases, Imperial College London, London, UK; 14 Medical Research Council Unit The Gambia at London School of Hygiene and Tropical Medicine, Atlantic Boulevard, Fajara, The Gambia; 15 The Francis Crick Institute, 1 Midland Road, London, NW1 1AT UK; 16 Division of Infection and Immunity, University College London, Gower Street, London, WC1E 6BT UK; 17 Department of Infectious Diseases, University College London Hospital, Euston Road, London, NW1 2BU UK; 18 Department of Clinical Sciences and International Public Health, Liverpool School of Tropical Medicine, Liverpool, UK; 19 Institut Pasteur, Université Paris Cité, Unité d’Épidémiologie Des Maladies Émergentes, Paris, France; 20 Service d’hépato-Gastroentérologie, CHU Yalgado OUÉDRAOGO, Université Joseph KI-ZERBO, Ouagadougou, Burkina Faso; 21 Malawi Liverpool Wellcome Trust Clinical Research Programme, Blantyre, Malawi; 22 Department of Clinical Infection, Microbiology and Immunity, University of Liverpool, Liverpool, UK; 23 Division of Infectious Diseases, Department of Medicine, Tygerberg Hospital and Stellenbosch University, Cape Town, South Africa; 24 School of Medicine, University of Alabama at Birmingham, Birmingham, AL USA; 25 Centre for Infectious Disease Research in Zambia, Lusaka, Zambia; 26 School of Medicine, University of Zambia, Lusaka, Zambia; 27 Department of Infectious Diseases, Bern University Hospital, University of Bern, Bern, Switzerland; 28 Department of Medicine, Jos University Teaching Hospital, Jos, Nigeria

**Keywords:** Hepatitis B, Africa, Advocacy, Prevention, Treatment

## Abstract

There are 82 million people living with hepatitis B (PLWHB) in the World Health Organization Africa region, where it is the main cause of liver disease. Effective vaccines have been available for over 40 years, yet there are 990,000 new infections annually, due to limited implementation of hepatitis B birth dose vaccination and antenatal tenofovir prophylaxis for highly viraemic women, which could eliminate mother-to-child transmission. Despite effective and cheap antiviral treatment which can suppress hepatitis B virus replication and reduce the risk of hepatocellular carcinoma (HCC), < 2% of PLWHB are diagnosed, and only 0.1% are treated. As a result, PLWHB are frequently diagnosed only when they have already developed decompensated cirrhosis and late-stage HCC, and consequently 80,000 hepatitis B-associated deaths occur each year. Major barriers include complex treatment guidelines which were derived from high-income settings, lack of affordable diagnostics, lack or insufficient domestic funding for hepatitis care, and limited healthcare infrastructure. Current treatment criteria may overlook patients at risk of cirrhosis and HCC. Therefore, expanded and simplified treatment criteria are needed. We advocate for decentralized community treatment programmes, adapted for low-resource and rural settings with limited laboratory infrastructure. We propose a strategy of treat-all except patients fulfilling criteria that suggest low risk of disease progression. Expanded treatment represents a financial challenge requiring concerted action from policy makers, industry, and international donor agencies. It is crucial to accelerate hepatitis B elimination plans, integrate hepatitis B care into existing healthcare programmes, and prioritize longitudinal and implementation research to improve care for PLWHB.

## Hepatitis B in Africa

Hepatitis B (HBV) is endemic in Africa, where an estimated 82 million people are chronically infected, with a prevalence of 6.1% (95% uncertainty interval 4.6–8.5) [1]. Despite safe and robust HBV vaccines being available for more than 40 years and highly effective nucleos(t)ide antivirals, an estimated 990,000 [95% UI 660,000–600,000] new HBV infections and 80,000 [95% UI 47,000–110,000] HBV-related deaths occurred in 2019 in the WHO Africa region [1].

HBV-related liver disease is compounded by 1.9 million people living with HIV-HBV and 1.6 million [95% CI 1.1–2.5] living with hepatitis D-HBV co-infections [2]. These co-infections accelerate the risk of HBV-related cirrhosis and hepatocellular carcinoma (HCC) [1, 3–5]. Increasing alcohol consumption and metabolic risk factors in Africa may further impact HBV outcomes in Africa as observed in developed countries.

Although 28 countries in the World Health Organization (WHO) Africa region have National Viral Hepatitis Strategic Plans, access to affordable and funded diagnostics, particularly point-of-care (POC) tests for hepatitis B surface antigen (HBsAg) and HBV DNA quantification molecular assays, are limited [1]. Consequently, in the WHO Africa region, only 1.8 million [95% UI 1.4–2.5] people living with hepatitis B (PLWHB) know their status, and approximately 110, 000 [95% UI 51,000–130,000] have received treatment [1].

Failure to prevent, diagnose, and treat HBV is the major factor contributing to cirrhosis and HCC burden on the continent. The 2017 Global burden of disease (GBD) study confirmed that sub-Saharan Africa (SSA) has the highest cirrhosis-related age-standardized death rate in the GBD super-regions with 32.2 [95% UI 25.8–38.6] deaths per 100,000. This is almost double the global level of 16.5 [15.8–18.1], with HBV the leading causative factor [3]. 48.9% of cirrhotic deaths in Western Africa, 31.2% in Central Africa, 25.9% in Eastern Africa, and 21.9% in Southern Africa are HBV-related [3]. These figures may even be underestimated due to lack of robust death registries in most African countries and the high proportion of deaths at home.

HCC was the second leading cause of cancer deaths in men and third in women in WHO Africa in 2020, with an estimated 38,629 incident cases and 36,592 HCC-related deaths [4]. Ten African countries (Egypt, The Gambia, Guinea, Ghana, Liberia, Burkina Faso, Senegal, Guinea-Bissau, Mauritania, and Cape Verde) are amongst the 25 countries with the highest age-standardized incidence rates of HCC [5]. HBV-related HCC presents at a median age of 42 years, is often aggressive, and multifocal with vascular invasion at the time of presentation [6]. Palliative interventions and care, a limited commodity in SSA, is invariably the only management option [7]. Fewer than 5% of HCC present at Barcelona Clinic Liver Cancer (BCLC) stage A-B where curative interventions are possible [6]. Prediction models for the development of HCC help to identify individuals at risk of HCC and in need of surveillance but need validation in SSA [8].

Reducing the burden of HBV infections in Africa will require a massive increase of public awareness, diagnosis, and linkage to care. In this perspective, we discuss how we can disrupt the *status quo* and drive HBV elimination in Africa.

## Barriers to HBV care and advocacy for public awareness in Africa

Low levels of awareness of HBV and its complications pose a major barrier to HBV diagnosis, management, and prevention [9, 10]. Furthermore, HBV endemic regions frequently have suboptimal clinical and public health services, as is seen in the WHO Africa region [11]. Based on international dogma, at present, most low-income countries recommend a high threshold for treatment, usually based on a combination of clinical, laboratory, and imaging parameters, which may be combined into scores such as aspartate aminotransferase to platelet ratio index (APRI). However, waiting for clinical evidence of liver injury exposes PLWHB to unnecessary risks and sustains a reservoir of virus in the population, which may drive ongoing transmission.

Failure to understand the main routes of HBV transmission in Africa, i.e. that it is predominantly childhood acquired rather than an adult sexually acquired infection, promotes inherent stigmatization. Furthermore, it is a vaccine preventable and silent disease, presenting in adulthood with established complications of cirrhosis and HCC. This engenders a siloed healthcare approach where HBV is managed expectantly when complications arise, rather than as a continuum of both prevention and care starting with mother-to-child and childhood preventive care through to adult linkage to care for those who screen HBsAg positive. A holistic approach focusing on prevention and harm minimization, targeted information, outreach testing with peer support, and linkage to services also is required to provide care for diverse and complex health and social needs [12].

In general, HBV clinical research has been limited with inadequate involvement of real-world populations of high burden [9, 13]. Additionally, vulnerable groups including refugees, people who are incarcerated, sex workers, and the Lesbian, Gay, Bisexual, Transgender, and Queer (LGBTQ) communities are under-served by clinical services and poorly represented [14]. Stigma and discrimination impair care seeking, and where they exist, HBV care programmes are siloed, instead of viewing HBV as part of a ‘syndemic’ challenge of multiple overlapping health issues [12, 15–17]. To date, international aid has been dedicated to HIV, tuberculosis, and malaria further promoting a siloed approach and hindering funding of integrated pathways of care.

In comparison to other global infectious diseases, such as HIV, representation and advocacy for PLWHB is limited [18]. Hence, civil society organizations such as the LiveWell Initiative in Nigeria, Saafara Hépatites Sénégal, and The National Organization for People Living with Hepatitis B (NOPLHB) in Uganda play a key role in driving awareness, advocacy, and the implementation of infrastructures at community level [19, 20].

### Role of advocacy and representation in the roll-out of wider treatment

Contextualizing HBV treatment guidelines, within the beliefs, behaviours, experiences, and expectations of PLWHB in different settings, is crucial [18, 21]. Affected community insights are needed to develop education and messaging that are culturally and linguistically appropriate and have a wide audience, including PLWHB and their families, healthcare workers, policy makers, and funders [22]. One specific need is to understand the attitudes of individuals and communities towards a ‘test-and-treat’ strategy, which is increasingly debated amongst clinicians, scientists, and policymakers. We need to explore perceptions and potential acceptability of a more inclusive treatment approach amongst affected communities, as this will be fundamental to inform changes to guidelines and to provide insights into real-world strategies for deployment of wider therapy, as well as opportunities for PLWHB to influence and co-design both research programmes and clinical service provision [22, 23]. HBV ‘*champions*’ and peer supporters are required to enhance the profile of HBV, education, and tackle stigma. To date, progress has been made through sharing stories as with the Hepatitis B Foundation (‘HepBStories.Org’) and the World Hepatitis Alliance (‘Stories’) [24, 25]. The Women in Hepatitis Africa, a sister organization of LiveWell Initiative, has trained over 5000 African women from 18 countries as ‘*Hepatitis Champions*’. The impact is measurable, as for example in the Makoko community in Nigeria there is over 90% uptake of HBV birth dose vaccination by low literacy women for their babies, and improved overall health outcomes for mothers and infants (personal communication; Bisi Bright, LiveWell Initiative). PLWHB are key to driving change and highlighting the problems of HBV infection whilst also being uniquely placed to address the issue of stigma.

Developing culturally and regionally targeted communication strategies for HBV can enhance knowledge and reduce stigma. For example, in Africa, most acquire HBV at birth or in early childhood, yet many communication approaches highlight HBV as a sexually transmitted infection (STI). This inadvertently promotes stigma and undermines care-seeking behaviour, particularly in regions with high HIV prevalence. In these settings, the proposed integration of HBV care into existing HIV programmes would mean that PLWHB share services. The potential unanticipated consequence may be the incorrect validation of a predominant STI nature of HBV and may further enhance stigma and discrimination [26]. However, data suggests that treating more PLWHB has the positive consequence of engaging communities better and attenuating HBV nihilism [27]. Enhancing awareness, education, notably about the natural history and transmission risks of HBV, and advocacy are crucial to break down barriers to HBV diagnosis and treatment, and investment is required to support culturally appropriate messaging that reaches diverse populations, community leaders, healthcare workers, and policy makers.

## Improved HBV prevention strategies

Vaccination, the cornerstone of HBV prevention, has been available for more than 40 years.

Administration of the hepatitis B birth dose vaccine (BDV) within 24 h of delivery is an affordable and cost-effective strategy negating long-term HBV risk [28, 29]. The evidence to support this comes mainly from East Asia, but recent studies have confirmed the benefit of BDV in Africa [30–32]. China, once the epicentre of the HBV epidemic, is on track to raise an HBV-free generation. We should accomplish this in African populations too.

Several WHO regions have achieved the 2020 Sustainable Development Goal of reducing HBV prevalence in children younger than 5 years to less than 1%. However, only 30% of countries in the WHO Africa region have adopted policies for universal BDV, and fewer than 10% of African infants receive timely BDV. There are challenges to rolling out BDV in Africa [33, 34] but possibly the most important one is lack of financial support from international stakeholders, like Gavi [35]. Gavi, recognizing the important impact of HBV BDV in Africa, committed to provide support for the introduction of HBV BDV from 2021 under their 2018 vaccine investment strategy [36]. In 2020, this was deferred due to the impact of the SARS-CoV-2 pandemic, but Gavi has now re-committed to the implementation of its HBV BDV programme [37–39].

Nevertheless, vaccination alone is insufficient to prevent transmission from highly viraemic and infectious mothers. Antiviral therapy administered from 28 weeks of pregnancy in women with HBV DNA levels > 200,000 IU/ml further reduces the transmission risk [40–43]. Coverage of antiretroviral therapy to prevent mother-to-child transmission (MTCT) of HIV in Africa increased from 44% in 2010 to 84% in 2018 [44]. Equal access to therapy should be available to HBV mono-infected women by leveraging existing perinatal prevention programmes [45]. Yet, less than 1% of pregnant women are screened and treated to prevent HBV MTCT in Africa, one of the main obstacles being the limited access to HBV viral load measurements.

The WHO triple elimination initiative (HIV, syphilis and HBV) ensuring universal access to affordable POC screening, treatment, and follow-up of all women for these important infections is thus key [46]. Extending screening (alongside treatment and/or vaccination if indicated) to partners and families is important to break cycles of infection within communities.

## Improved access to HBV diagnostics

In HBV care, molecular diagnostics are often more expensive than the costs of 1 year of treatment. Prevention strategies and expanded treatment access programmes can only be realized if affordable and reliable diagnostics are available. This includes both POC assays for selected laboratory analyses, tools for the assessment of liver fibrosis, and biomarkers of disease progression and HCC risk. Fibrosis assessment tools currently recommended in international liver society guidelines, such as Fibroscan®, are prohibitively expensive and probes must often be shipped annually to Europe for calibration [47].

WHO prequalified HBsAg POC tests must be available at primary care level to identify and link PLWHB to care. HBV DNA POC testing has the potential to risk-stratify PLWHB and reduce the delay in initiation of antiviral therapy. Hepatitis B core-related antigen (HBcrAg) POC rapid tests, a surrogate for a high HBV viral load, were evaluated in The Gambia and demonstrated sensitivity of 73% and specificity of 92% for HBV diagnosis using the cutoff of DNA level > 2000 IU/ml and sensitivity of 91% and specificity of 86% when using > 200,000 IU/ml as cutoff [48]. Similarly, HBcrAg has been shown to be a good predictor of HBeAg seroconversion [49] and HCC incidence [50].

Better community screening initiatives are also necessary to raise awareness of viral hepatitis. Linking HBV screening to other events (e.g. census initiatives and village festivals) could be novel strategies that would afford many people an opportunity for testing. This strategy has been employed successfully in Rwanda for hepatitis C (HCV) screening [51, 52]. Cooperation with religious leaders with the linkage of campaigns and public education with major religious festivals may also aid in raising awareness and testing rates. Egypt is an outstanding example of how a national screening campaign (for HCV) can cover the whole population in a short period—which can be a model for other African countries [53].

## Simplified and expanded access to HBV treatment

Based on guidance to date, most of those diagnosed with chronic HBV infection do not meet the current criteria for treatment, resulting in the initiation of therapy being withheld (e.g. < 10% eligibility in a study in The Gambia) [54]. This concept of requiring regular visits to healthcare providers for monitoring purposes without receiving any treatment may not be adequately explained or easily understandable for PLWHB who did not meet treatment criteria. Consequently, PLWHB may seek alternative interventions and choose traditional medicines, which are potentially hepatotoxic, in the hope of cure, often delaying or inhibiting optimal care-seeking.

The nucleoside analogues tenofovir disoproxil fumarate (TDF) and entecavir suppress hepatitis B virus replication, prevent and reverse liver fibrosis, reduce the incidence of HCC, improve patient-reported quality of life, and reduce HBV transmission [55–59]. Excellent long-term safety data are reported, including for TDF in pregnancy [21, 60, 61]. A low risk of emergent drug resistance has been observed after long-term therapy with TDF due to a high genetic barrier to resistance [21, 62]. TDF has a small risk of renal dysfunction and metabolic bone disease, and risk is highest in those with pre-existing renal disease or diabetes; an alternative formulation (tenofovir alafenamide, TAF) or entecavir can be used for patients with specific risk factors [59, 63]. TDF, particularly as a combination treatment with lamivudine or emtricitabine, is widely available in the WHO Africa region, and is frequently used as a component of HIV treatment and for pre-exposure prophylaxis (PrEP). TDF is low-cost, with a suggested benchmark pricing of $2.63 USD per month, although there is large regional variation [64]. TDF in combination with lamivudine or emtricitabine can also be used to treat chronic HBV and potentially increases access to therapy. Novel therapeutics, including agents targeting a functional cure, are in development [65, 66]. When available, early and affordable access to these therapies for PLWHB in endemic regions should be a priority and African centres should be included in clinical trials.

### The treatment gap: barriers to life-saving treatment

Of 82 million PLWHB in Africa, an estimated 19% meet the WHO 2015 treatment criteria; however, less than 2% have been diagnosed, and only 0.1% of all PLWHB are on treatment [67, 68]. This treatment gap represents millions of PLWHB who potentially would benefit from antiviral therapy now and who are at risk of progression to cirrhosis or HCC. Major barriers to antiviral therapy include the following: limited case-finding, linkage to and delivery of sustainable care, and the complexity of current treatment guidelines which recommend repeated clinical assessments that are difficult to implement in practice for assessing treatment eligibility [59, 69, 70]. There is also lack of longitudinal data on retention in care of PLWHB in Africa [71]. APRI (AST to Platelet Ratio Index) is a recommended non-invasive test to assess the likelihood of fibrosis or cirrhosis and guide initiation of antiviral therapy. However, recent data from the HEPSANET cohort demonstrated that the WHO-recommended APRI treatment threshold of 2.0 for the diagnosis of cirrhosis was too high and was associated with a low sensitivity for diagnosing cirrhosis, relative to a transient elastography reference standard [72]. Lowering the APRI threshold to 0.65 was associated with improved sensitivity with only marginal reduction in specificity. Notably, this new threshold has already been implemented in national guidelines in Ethiopia and Malawi [73]. Overall, APRI is a tool with reasonable discriminant performance albeit an imperfect classifier of liver fibrosis. Any selected threshold represents a trade-off between maximizing sensitivity to avoid missing significant liver fibrosis and maximizing specificity which could create excessive burdens on health systems from treatment needs and risk of life-long therapy to those who may not need it [72].

HBV case-finding, treatment, and monitoring programmes in the WHO Africa region are under-resourced. Thus, for many PLWHB, their first presentation is with clinical manifestations of liver disease [74]. Even with a simplified strategy for settings without access to HBV DNA quantification, where ALT monitoring is used as the major criterion for treatment, repeated clinic visits are required before deciding on treatment. Monitoring intervals may also be associated with insufficient precision to identify clinically significant liver inflammation and may lead to significant loss to follow-up [21]. In Sierra Leone, amongst untreated patients who were ineligible for treatment on initial assessment, fewer than one third were retained in care at 1 year [75]. This can be contrasted with the simplicity for clinicians and patients of the treat-all paradigm for HIV and HCV. In a pilot HBV treatment programme in Ethiopia, one of the primary reasons for delay in commencement of treatment was the lack of access to HBV DNA quantification, according with similar experiences reported from Sierra Leone [75, 76]. The need for community screening cannot be over emphasized as this will help reduce the limited case finding and late presentations, due to early diagnosis.

### The case for simplified and expanded treatment criteria

Accumulating evidence from observational studies suggests that certain categories of individuals, who are ineligible for treatment according to the conventional treatment eligibility criteria, may still develop cirrhosis and HCC [21, 77]. For instance, in South Korea, an observational study suggested that HBV-infected individuals with HBV DNA levels of ≥ 2000 IU/mL, who never met treatment eligibility criteria, had a higher risk of developing HCC compared to the propensity-matched treated cohort that achieved virological response with a nucleos(t)ide analogue [77]. Similarly, in an observational study in USA and in Taiwan, antiviral therapy was associated with reduced HCC risk in patients with ALT < 2 × upper limit of normal (ULN), particularly when they had HBV DNA levels of ≥ 2000 IU/mL [78]. In another South Korean study, patients with elevated HBV DNA levels and normal to mildly elevated ALT had a significantly increased risk of HCC or death or liver transplantation compared to those treated for ALT ≥ 2 ULN in a propensity score-matched analysis [79]. With early infection in the HBeAg positive chronic infection phase without significant ALT elevation, genomic integration and clonal hepatocyte expansion are critical events that drive hepatocarcinogenesis [80]. Given imperfect monitoring schedules and significant loss to follow-up even in well-resourced settings, inadequate surveillance may also lead to an unrecognized transition to a higher risk phenotype [81].

Observational studies of HIV/HBV co-infected patients in South Africa, USA, and Hong Kong have reported significantly lower incidences of HCC, relative to HBV mono-infected patients. This is potentially more attributable to universal treatment for HIV, rather than implementation of selective criteria for HBV [82–84]. In South Africa, HBV/HIV co-infected patients were more likely to have had liver fibrosis assessment and to be on antiviral therapy, whereas HBV monoinfected patients had a higher prevalence of liver disease, highlighting disparities in care relative to provision for people living with HIV [84]. Within the HEPSANET study, many patients fall into indeterminate phenotypes that do not fulfil the classifications in the EASL guidelines and would not be recommended for treatment with existing criteria [85]. For example, 360 (11.8%) of the cohort were HBeAg negative with HBV DNA > 2000 IU/ml but normal ALT [85]. Long-term outcomes amongst this group are unknown.

The natural history of HBV infection in Africa is inadequately characterized. In East, Southern, and West Africa, genotypes A, D, and E are endemic, relative to the Western Pacific region where much of our understanding of the natural history and determinants of disease progression are derived and where genotypes B and C predominate [86]. Inherent genetic determinants of immune control and immunopathogenesis of HBV are likely to differ as is exposure to hepatotoxic agents such as aflatoxins or immune modulating exposures such as malaria, helminths, schistosomiasis, and HIV. Although a longitudinal follow-up of PLWHB in The Gambia found that persistently elevated HBV DNA levels ≥ 2000 IU/mL or ALT ≥ 40 IUL was associated with an increased risk of significant fibrosis after a median follow-up of 28 years [87], at present in Africa, there is limited data to adequately inform risk stratification of patients compared to Asia.

## Rationale for a ‘Treat-all-except’ strategy

Given the urgency required to narrow the treatment gap, a realistic approach is to optimize the use of existing, imperfect diagnostic tests, with a focus on those that can be rapidly integrated and scaled up within routine health services in decentralized healthcare facilities in Africa. One approach could be instead of identifying patients with a high probability of disease progression based on data extrapolated from Asian or European studies, to instead identify patients at low risk and treat the remainder; a ‘Treat-all-except’ paradigm. In our HEPSANET study, we estimated that an optimized rule-out threshold of 0.36 would increase the sensitivity of the APRI score for diagnosis of cirrhosis to 80.6% (76.1–85.1%) with a specificity of 64.3% (62.8–65.8%) [72]. This would lead to a large increase in the treatment-eligible population, whilst still being more selective than a ‘treat-all’ approach which has been proposed for HBV [21, 88]. However, in the African setting where less than 2% of PLWHB have been diagnosed, an active ‘screen all’ public health programme is required for a ‘Treat-all-except’ strategy to have an impact on overall disease burden. This further emphasizes the need for affordable and well performing POC diagnostics to be upscaled and accepted by healthcare workers as a means to screen (Table 1).

**Table 1:** Expanded access to treatment in the WHO Africa region: The arguments for and against

Consideration	Arguments for an inclusive approach (‘Treat-all-except’)	Arguments for a restrictive/traditional approach (‘treat only if…’)
**Financial implications**	Potential long-term cost-effectiveness, based on reduction of complications and transmission	Avoid potentially unnecessary treatment that may represent an excessive financial impact on healthcare systems with conflicting priorities and scarce resources Expanded treatment may not be cost-effective in all settings
**Retention in care**	Avoid the need for HBV DNA testing with its inherent complexities (stock-outs, out of pocket costs, capital and maintenance costs) in resource-limited settings. May enhance the initiation of antiviral treatment which is associated with improved retention in care, relative to deferral and monitoring	Investigations such as HBV DNA and ALT may reduce the uptake of initiating antiviral therapy; but can help patients to understand their disease stage, and therefore may enhance the retention in care for those initiated
**Risk of liver complications in low-risk groups**	A more inclusive model might be pertinent in Africa, given limited natural history data from the region and the high incidence of HBV-related cirrhosis	A more restrictive model reflects the uncertain benefit of treatment for patients with normal ALT and low HBV DNA, and reduces exposure to unnecessary costs and side-effects
**HCC incidence**	Young age of HCC and poor prognosis at time of diagnosis in real-world cohort studies from Africa	Age-standardized HCC incidence do not show an excess of cases for WHO Africa; age of onset might be explained by population age distribution
**HBV transmission**	Expanded treatment access reduces risk of horizontal and vertical transmission	HBV DNA quantification can inform transmission risk
**Longitudinal outcome data**	Less loss to follow up in treated compared to untreated populations	Longitudinal monitoring of untreated patients can refine and optimize treatment criteria specific to WHO Africa

*Abbreviations*: *ALT* alanine transaminase, *DNA* deoxyribonucleic acid, *HBV* hepatitis B, *HCC* hepatocellular carcinoma, *WHO* World Health Organization

### A public health perspective

HBV programmes should apply lessons learned from HIV and HCV care to simplify treatment and monitoring guidelines and adopt task sharing and decentralized models of treatment [89]. In addition, management of HBV can be regarded as a primary prevention approach analogous to treating risk factors for cardiovascular disease (e.g. hypertension) to reduce the risk of adverse events in the long term [21].

Some countries have already introduced treatment guidelines that are much less selective than the present recommendations from the WHO 2015 or International Hepatology Society Guidelines [59, 69, 70, 90]. China has recently published a simplified HBV treatment algorithm where anyone over the age of 30 is eligible for treatment if they are HBsAg positive with detectable HBV DNA. Anyone younger than age 30 with detectable HBV DNA is eligible for treatment if they have compensated cirrhosis, fibrosis stage ≥ F2, ALT > ULN, family history of cirrhosis or HCC, or extrahepatic manifestations [91]. A modelling study from China found that the most cost-effective approach strategy capable of meeting the WHO 2030 target of 65% reduction in mortality was to treat those aged 18–80 years with HBsAg-positive disease, regardless of ALT values, aiming for 80% coverage [92]. Importantly, they estimated that cost-effectiveness would be inversely proportional to the time taken to implement such changes [92], highlighting the need for action.

HBV mono-infected individuals often struggle to access TDF in Africa. In contrast, HIV PrEP programmes have been scaled up for widespread provision of prophylactic antiviral therapy (e.g. TDF/FTC, Truvada^R^) to otherwise healthy people with excess HIV risk, thereby enabling over one million people to access PrEP in Africa [93, 94].

Access to and the provision of PrEP regimens (TDF/FTC, Truvada^R^), which are generally more available and affordable within HIV-funded programmes throughout Africa for PLWHB, could enable an opportunity for the provision of integrated HBV care and increased access to HBV therapy within existing HIV programmes. An additional benefit of HBV-active HIV PrEP would be protection against HIV in high prevalence regions. This will require increased investment in the infrastructure required to manage the additional caseload of patients with HBV-infection on already stretched HIV clinical services. The funding implications of this approach would require re-addressing international and national funding of infectious disease programmes. The Global Fund to fight AIDS, TB, and Malaria (GFATM) policies have now evolved to enable countries to request more resources to support viral hepatitis, harm reduction and triple elimination services. However, a potential risk to this approach is increasing stigma associated with hepatitis B as PrEP is associated with HIV. Implementation research will be essential to ensure services are accessible and acceptable to PLWHB.

### Implementation: challenges and opportunities

The financial impact of expanded HBV treatment represents a challenge for national health services, international donor agencies, and individual citizens, depending on country-level HBV funding arrangements. An analysis of the REVEAL-HBV cohort from USA and Taiwan demonstrated that the number of people needed to treat could be over 100 for prevention of HCC in those with ALT levels < 2 × ULN and HBV DNA < 2000 IU/ml [78]. However, there may be other potential benefits at individual and population-level, including reduced transmission, improved quality of life, reduced incidence or reversal of cirrhosis, and improved retention in care. Cost-effectiveness analyses of treatment that represent African settings are urgently needed. Ensuring retention in care and adherence to treatment will be especially critical in an asymptomatic population, for a subset of whom the benefit is uncertain.

## Call to action and future directions in HBV elimination

Seven years have passed since the World Health Assembly sanctioned the elimination plan for viral hepatitis, but little progress has been made in the WHO Africa region [1]. The consequences are evident: two thirds of new HBV infections globally occur in Africa, less than 1% of those eligible for treatment in Africa are treated, and HBV-related HCC is predicted to rise [95]. Concerted action from policy makers, academia, and industry is urgently needed to reach the elimination goals. Experiences from HIV/AIDS have shown how it is possible to elicit change if all stakeholders work together. We recommend several action points involving policy makers, industry and academia as well as research gaps that are of the highest priorities in our opinion (Table 2).

**Table 2:** Call to action and future directions in HBV elimination

*Policy makers (National Ministries of Health (NMoH), Gavi, Africa CDC, WHO)*
**Prevention of mother-to-child transmission** • NMoH must ensure access to birth dose HBV vaccine • Gavi should support countries with birth dose HBV vaccine • HBV DNA needs to be made universally available to assess need for TDF prophylaxis and to monitor ongoing antiviral therapy • Pregnant women need access to education, diagnosis and risk-stratification • Antiviral prophylaxis must be available in pregnancy when HBV DNA is high
**Set up national treatment programs for HBV** • NMoH cannot wait for external funding. Treatment of HBV is cost-effective and should be an urgent priority [96].
**African HBV treatment guidelines** • Africa CDC should take the lead in driving the development of African Guidelines • Given the diversity of healthcare services in Africa, guidelines cannot take a one-size-fits-all approach but should be tiered to available resources (e.g. with or without access to HBV DNA) • Lack of laboratory facilities should not disqualify patients from treatment • We propose a new paradigm in HBV therapy in LMICs: ‘Treat-all-except’. This approach will ensure that most patients with or at high risk of significant liver disease are treated and could save costs compared to a ‘Treat-all’ strategy
**Integration, simplification, task sharing, decentralization** • These were success factors in HIV programs and NMoH do not need to reinvent the wheel when setting up HBV care and treatment services • Integration will further promote triple elimination goals for HIV, syphilis, and hepatitis B • Simplified treatment guidelines with task sharing from physicians to nurses/health officers and decentralization outside specialized hospitals • Training of peer supporters and community advocacy is essential
**Awareness, screening, and linkage-to-care** • NMoH must prioritize active screening campaigns; reliance on passive case finding will not have significant public health impact For hepatitis B, screening might be done only once for the whole adult population; there is no need for repeated mass screening in countries where transmission mainly occurs in childhood
***Industry*** • ***Access to drugs****:* Although TDF is off-patent, access for HBV mono-infected patients is still restricted in many countries since it is only registered as an HIV drug. Pharmaceutical companies must take on this responsibility and remove bureaucratic obstacles to scaling up HBV treatment in Africa. Affordable access to Entecavir and TAF is also important in PLWHB who have renal impairment or are at risk of osteoporosis • ***Access to diagnostics:***Industry should be prompted to set up access programmes in LMICs for affordable and reliable POC assays for HBsAg and HBV DNA viral load testing; and transient elastography machines. The HIV sector managed to develop a range of POC assays for CD4 cell counts and HIV viral load [97]; these technologies could be modified to test for HBV DNA. It is essential to validate tools that can be utilized in decentralized settings. For instance, investing in the improvement and prequalification of a rapid test for hepatitis B e antigen is an area deserving attention
***Academia*** • ***Share data from Africa:*** HBV data from Africa are scarce, and clinicians/researchers on the continent working with PLWHB should be encouraged to share their data, preferably via larger registries like the *HEPSANET* collaboration (https://www.hepsanet.org or Research platforms such as PROLIFICA (https://www.prolifica.africa/team-partners/the-team) • ***Prioritize high-impact studies:*** There are urgent research gaps that need to be prioritized to inform guidelines and influence policy in Africa. These include: ◦ Large longitudinal cohort studies to highlight the following: (i) the natural history in untreated patients, (ii) risk factors for HCC and cirrhosis, (iii) optimal threshold for initiating antiviral therapy, and (iv) treatment efficacy, resistance, and long-term toxicity ◦ Studies on the feasibility and cost-effectiveness of different models of care: (i) Treat-all, (ii) Treat-all except…, (iii) Treat only if… ◦ Study strategies to integrate HBV testing and treatment into existing healthcare systems ◦ PMTCT implementation research as part of the WHO triple elimination strategy ◦ Surveillance strategies and low-cost diagnostic markers for HCC

*Abbreviations*: *Africa CDC* Africa Centres for Disease Control and Prevention, *DNA* deoxyribonucleic acid, *HCC* hepatocellular carcinoma, *HBV* hepatitis B, *HEPSANET* Hepatitis B in Africa Collaborative Network, *HIV* human immunodeficiency virus, *NMoH* National Ministries of Health, *PLWHB* People living with Hepatitis B, *PMTCT* prevention of mother-to-child transmission, *POC* point-of-care, *PROLIFICA* Prevention of Liver Fibrosis and Cancer in Africa, *WHO* World Health Organization

## Conclusions

Maintaining the status quo in the management of hepatitis B in Africa is intolerable. In order to prevent new infections, HBV BDV must be made available for all newborns in Africa. The current model that involves repeated laboratory assessments and complex criteria for the initiation of antiviral therapy together with the cost of diagnostics is an obstacle to the identification of those PLWHB requiring therapy. There is a stark lack of education and awareness, poor access to relevant laboratory tests and imaging, and inconsistent access to appropriate medication. This is particularly pertinent in resource-constrained settings. HBV natural history data from Africa are scarce, and clinicians and researchers on the continent working with PLWHB should be encouraged to share and publish their data within strong clinical governance structures for data sharing such as the HEPSANET collaboration so that guidance documents can be based on longitudinal cohort studies that address the long-term outcomes of chronic hepatitis B as well as the cost-effectiveness and feasibility of different models of care in Africa.

Decentralization, integration of services, and task-sharing with adequate funding of the required infrastructure will be key to upscaling delivery of care for PLWHB. However, new approaches such ‘Treat all’ or ‘Treat-all-except’ approaches do need further evidence from implementation science research, including qualitative evaluation of understanding, attitudes, and acceptability of such approaches by both PLWHB and Ministries of Health.

## Data Availability

Not applicable.
